# Supplementary material for: Evaluation of Salivary Biomarkers of Periodontal Disease Based on Smoking Status: A Systematic Review
Source: Int J Environ Res Public Health. 2022 Nov 7;19(21):14619. doi: 10.3390/ijerph192114619 (PMC9657317; doi:10.3390/ijerph192114619)
Supplement: Supplementary file 1 [file ijerph-19-14619-s001.zip › Table S1.pdf]

# Supporting Information

## Table S1. Search strategy

| Plain text / natural language | Controlled vocabulary (MeSH) |
|---|---|
| **Terms for target condition: periodontal disease** | |
| Periodontal diseas*<br>Parodontos*<br>Pyorrhea Alveolaris | Periodontal disease |
| Furcation Defect | Furcation Defects |
| Gingival Diseas*<br>Epuli*<br>Gingivos* | Gingival Diseases |
| Gingival Hemorrhag* | Gingival Hemorrhage |
| Gingival Neoplas*<br>Congenital Epuli* | Gingival Neoplasms |
| Gingival Overgrowt* | Gingival Overgrowth |
| Gingival Hypertroph* | Gingival Hypertrophy |
| Gingival Hyperplasi* | Gingival Hyperplasia |
| Gingival Fibromatos*<br>Fibromatosis Gingivae | Fibromatosis, Gingival |
| Gingival Recessio*<br>Gingival Atroph*<br>Atrophy of Gingiva<br>Gingiva Atroph* | Gingival Recession |
| Gingiviti*<br>Gingival inflammation | Gingivitis |
| Vincent Infection<br>Vincent's Infection<br>Fusospirillosis<br>Vincent's Stomatitis<br>Phagedenic Gingivitis<br>Vincent's Gingivitis<br>Trench Mouth<br>Vincent Angina<br>Ulcerative Stomatitis | Gingivitis, Necrotizing Ulcerative |
| Gingival Pocke* | Gingival Pocket |
| Peri Implantiti*<br>Peri-Implantiti*<br>Periimplantiti* | Peri-Implantitis |
| Periapical | Periapical Diseases |
| Dental Granulom* | Periapical Granuloma |
| Periapical Absces*<br>Apical Alveolar Absces*<br>Dentoalveolar Absces* | Periapical Abscess |
| Periodontal Atrophies<br>Atrophy of Periodontium<br>Periodontium Atroph*<br>Gingivo-Osseous Atroph* | Periodontal Atrophy |
| Periodontal Attachment Loss | Periodontal Attachment Loss |
| Gingival Recessio*<br>Gingival Atroph*<br>Atrophy of Gingiva<br>Gingiva Atroph* | Gingival Recession |

| Plain text / natural language | Controlled vocabulary (MeSH) |
|---|---|
| Alveolar Bone Los*<br>Alveolar Process Atroph*<br>Alveolar Resorptio*<br>Periodontal Bone Los*<br>Periodontal Resorptio*<br>Alveolar Bone Atroph* | Alveolar Bone Loss |
| Periodontal Cys*<br>Dental Root Cys*<br>Dentoalveolar Cys*<br>Lateral Cys* | Periodontal Cyst |
| Radicular Cys*<br>Periapical Cys*<br>Apical Periodontal Cys* | Radicular Cyst |
| Periodontiti*<br>Pericementiti* | Periodontitis |
| Aggressive Periodonti*<br>Circumpubertal Periodontitis<br>Prepubertal Periodontitis<br>Early-Onset Periodontiti*<br>Early Onset Periodontiti*<br>Juvenile Periodontiti*<br>Periodontos* | Aggressive Periodontitis |
| Chronic Periodontiti*<br>Adult Periodontiti* | Chronic Periodontitis |
| Periapical Periodontiti*<br>Apical Periodontiti*<br>Acute Nonsuppurative Periodontiti*<br>Periapical Absces*<br>Apical Alveolar Absces*<br>Apical Dentoalveolar Absces*<br>Periapical Granulom*<br>Dental Granulom* | Periapical Periodontitis |
| Periodontal Pocke* | Periodontal Pocket |
| Periodontal Absces* | Periodontal Abscess |
| Tooth Los* | Tooth Loss |
| Tooth Migration<br>Tooth Drif* | Tooth Migration |
| Teeth Mesial Movement<br>Mesial Migration of Teeth<br>Teeth Mesial Migration<br>Mesial Drift of Teeth<br>Teeth Mesial Drift | Mesial Movement of Teeth |
| Tooth Mobilit* | Tooth Mobility |
| Periodontal status<br>Periodontal Index<br>Periodontal Indices<br>Periodontal Indexes<br>Community Periodontal Index of Treatment Needs<br>CPITN<br>Gingival Bleeding on Probing<br>Gingival Index<br>Gingival Indices | Periodontal Index |
| Periodontal evaluation | |
| Stimulated saliva | |
| 1 or 2 or 3 or ~ or 134 or 135 or 136 | |
| **Terms for the type of oral sample analyzed: saliva** | |
| Saliv* | Saliva |
| 138 or 139 | |

| Plain text / natural language | Controlled vocabulary (MeSH) |
|---|---|
| **Terms for the index tests: salivary biomarkers** | |
| **Saliva Enzymes** | |
| ALP<br>alkaline phosphatase | Alkaline Phosphatase |
| ACP<br>Acid Phosphatase<br>Acid beta-Glycerophosphate<br>Acid β-glycerophosphate | Acid Phosphatase |
| AST<br>Aspartate Aminotransferase<br>Aspartate Transaminase<br>Glutamic-Oxaloacetic Transaminase<br>Glutamic Oxaloacetic Transaminase<br>L-Aspartate-2-Oxoglutarate Aminotransferase<br>L Aspartate 2 Oxoglutarate Aminotransferase<br>Glutamate-Aspartate Transaminase<br>Glutamate Aspartate Transaminase<br>SGOT<br>Serum Glutamic-Oxaloacetic Transaminase<br>Serum Glutamic Oxaloacetic Transaminase | Aspartate Aminotransferases |
| ALT<br>Alanine Transaminase<br>Glutamic-Alanine Transaminase<br>Glutamic Alanine Transaminase<br>Alanine-2-Oxoglutarate Aminotransferase<br>Alanine 2 Oxoglutarate Aminotransferase<br>ALAT<br>Alanine Aminotransferase<br>SGPT<br>Glutamic-Pyruvic Transaminase<br>Glutamic Pyruvic Transaminase<br>Glutamate-Pyruvate Transaminase<br>Glutamic-Pyruvic Transaminase | Alanine Transaminase |
| LDH<br>L-Lactate Dehydrogenase A<br>LDH-A<br>LDHA<br>L-Lactate Dehydrogenase B<br>LDH-B<br>LDHB<br>L-Lactate Dehydrogenase C<br>LDH-C<br>LDHC<br>L-Lactate Dehydrogenase<br>Lactate Dehydrogenase | L-Lactate Dehydrogenase |
| CRE<br>Creatinine | Creatinine |
| BUN<br>Blood Urea Nitrogen | Blood Urea Nitrogen |
| UA<br>Urea | Urea |
| F-Hb<br>Free-hemoglobin | |
| Neopterin<br>2-Amino-6-(1,2,3-trihydroxypropyl)-4(3H)-pteridinone<br>Monapterin | Neopterin |

| Plain text / natural language | Controlled vocabulary (MeSH) |
|---|---|
| MDA<br>Malondialdehyde<br>Propanedial<br>Malonyldialdehyde<br>Malonaldehyde<br>Malonylaldehyde | Malondialdehyde |
| GSHPx<br>Glutathione Peroxidase<br>Lipoperoxidase | Glutathione Peroxidase |
| SOD<br>Superoxide dismutase<br>Erythrocuprein<br>Hemocuprein | Superoxide Dismutase |
| MMP-8<br>MMP8<br>Matrix Metalloproteinase-8<br>Matrix Metalloproteinase 8<br>Neutrophil Collagenase<br>Fibroblast Collagenase<br>Collagenase-2<br>Collagenase2 | Matrix Metalloproteinase 8 |
| MMP-9<br>MMP9<br>Matrix Metalloproteinase-9<br>Matrix Metalloproteinase 9<br>92-kDa Type IV Collagenase<br>92 kDa Type IV Collagenase<br>92-kDa Gelatinase<br>92 kDa Gelatinase<br>Gelatinase B | Matrix Metalloproteinase 9 |
| TIMP-1<br>TIMP1<br>Tissue Inhibitor of Metalloproteinase-1<br>Tissue Inhibitor of Metalloproteinase 1 | Tissue Inhibitor of Metalloproteinase-1 |
| MPO<br>Myeloperoxidase<br>Peroxidase | Peroxidase |
| IL-1β<br>Interleukin-1beta<br>Interleukin 1beta<br>IL-1 beta<br>Interleukin-1 beta<br>Interleukin 1 beta<br>Catabolin | Interleukin-1beta |
| IL-6<br>Interleukin-6<br>IL6<br>B-Cell Stimulatory Factor-2<br>B-Cell Differentiation Factor-2<br>BSF-2<br>Hybridoma Growth Factor<br>IFN-beta 2<br>Plasmacytoma Growth Factor<br>Hepatocyte-Stimulating Factor<br>MGI-2<br>Myeloid Differentiation-Inducing Protein<br>B-Cell Differentiation Factor<br>Interferon beta-2 | Interleukin-6 |

| Plain text / natural language | Controlled vocabulary (MeSH) |
|---|---|
| IL-8<br>IL8<br>Interleukin 8<br>Interleukin-8<br>Monocyte-Derived Neutrophil Chemotactic Factor<br>Neutrophil Activation Factor<br>Lymphocyte-Derived Neutrophil-Activating Peptide<br>Monocyte-Derived Neutrophil-Activating Peptide<br>Alveolar Macrophage Chemotactic Factor-I<br>AMCF-I<br>Anionic Neutrophil-Activating Peptide<br>Chemokine CXCL8<br>Macrophage-Derived Chemotactic Factor<br>Neutrophil Chemotactic Factor | Interleukin-8 |
| TNF-a<br>Tumor Necrosis Factor Blockers<br>TNF Inhibitors<br>TNF Blockers<br>Tumor Necrosis Factor Blocker<br>Tumor Necrosis Factor Antagonist<br>Tumor Necrosis Factor Inhibitor<br>TNF Antagonist<br>TNF Blocker<br>TNF Inhibitor<br>Tumor Necrosis Factor Antagonists<br>TNF Antagonists<br>Tumor Necrosis Factor-a | Tumor Necrosis Factor Inhibitors |
| Lysozyme<br>N-Acetylmuramide Glycanhydrolase | Muramidase |
| Leukocyte Elastase<br>Neutrophil Elastase<br>Polymorphonuclear Leukocyte Elastase<br>Neutrophil Elastase<br>PMN Elastase<br>Granulocyte Elastase<br>Lysosomal Elastase | Leukocyte Elastase |
| Lymphocyte<br>Lymphoid Cell | Lymphocytes |
| Uric Acid<br>Trioxopurine<br>Urate | Uric Acid |
| 8-iso PGF2α<br>8-isoprostaglandin F2a | Dinoprost |
| **< Integration concepts of Saliva Enzymes>**<br>141 or 142 or 143 or ~ or 318 or 319 or 320 | |
| **Immunoglobulin (Ig)** | |
| Immunoglobulin A<br>IgA | Immunoglobulin A |
| Immunoglobulin D<br>IgD<br>IgD1<br>IgD2 | Immunoglobulin D |
| Immunoglobulin E<br>IgE | Immunoglobulin E |
| Immunoglobulin G<br>IgG | Immunoglobulin G |
| Immunoglobulin M<br>IgM | Immunoglobulin M |
| **< Integration concepts of immunoglobulin>**<br>322 or 323 or 324 or ~ 336 or 337 or 338 | |

| Plain text / natural language | Controlled vocabulary (MeSH) |
|---|---|
| **Hormones** | |
| Cotinine<br>Scotine | Cotinine |
| Cortisol<br>Hydrocortisone | Hydrocortisone |
| **<Integration concepts of terms for hormones>**<br>340 or 341 or 342 or 343 or 344 or 345 | |
| **<Integration concepts of terms for the index tests>**<br>321 or 339 or 346 | |
| **Terms for target condition AND**<br>**Terms for the type of oral sample analyzed AND**<br>Terms for the index tests: salivary biomarkers<br>137 **AND** 140 **AND** 347 | |
| 348 **NOT** ("review"[Publication Type] **OR** "review literature as topic"[MeSH Terms]) | |
